# A Prospective Comparative Study of the Effectiveness of Flap Fixation by Suture Versus Conventional Closure in Reducing Seroma Formation After Modified Radical Mastectomy

**DOI:** 10.7759/cureus.83915

**Published:** 2025-05-11

**Authors:** Aghosh Raju, Swagata Brahmachari, Ajeet P Maurya, Mangalapalle MadhuBabu, Ananthakrishnan M.

**Affiliations:** 1 General Surgery, All India Institute of Medical Sciences, Bhopal, IND; 2 Community and Family Medicine, All India Institute of Medical Sciences, Bhopal, IND

**Keywords:** axillary exclusion, axillary lymph node dissection, breast cancer, flap fixation, modified radical mastectomy, post-mastectomy seroma

## Abstract

Background

Seroma formation is a common postoperative complication of modified radical mastectomy (MRM), leading to delayed wound healing, increased infection risk, and prolonged hospital stays. Flap fixation by suture has been proposed to reduce seroma incidence, but its efficacy remains to be established.

Methods

A prospective comparative study was conducted at a tertiary referral center in central India from April 2019 to March 2020, involving 72 patients undergoing MRM for breast cancer (BC). Patients were divided into two groups: the study group (n = 36) underwent flap fixation with axillary exclusion by fine interrupted absorbable sutures, while the control group (n = 36) underwent conventional wound closure. Outcomes assessed included seroma incidence, drain duration, total drain output, and postoperative morbidities such as pain, surgical site infection (SSI), and flap necrosis.

Results

Patients in the flap fixation with axillary exclusion group had significantly reduced total drain output (mean: 306.67 mL versus 531.11 mL, p<0.01) and earlier drain removal (mean: 4 days versus 6.25 days, p<0.001) compared to the control group. The incidence of seroma was significantly lower in the study group (11.1% versus 41.7%). Postoperative pain, flap necrosis, and SSI were comparable between the groups signifying that flap fixation with axillary exclusion does not increase the morbidity with acceptable cosmesis.

Conclusion

Flap fixation with axillary exclusion by suture effectively reduces seroma formation and accelerates recovery without compromising patient comfort or mobility, representing an improved technique for MRM closure.

## Introduction

Breast cancer (BC) has emerged as a major global health issue, being the most common cancer and a leading cause of cancer-related deaths in women. BC accounts for 11.7% of all cancer diagnoses worldwide and 13.5% of all cancers in India [1]. Management of BC involves a multidisciplinary approach, with surgery as one of the definitive options. Surgery for breast carcinoma includes breast-conserving surgery (BCS) and modified radical mastectomy (MRM) with or without reconstruction. With the advancement in surgical techniques, although BCS is preferred more, MRM is still the most commonly performed surgery for the definitive management of BC [2].

Postoperative seroma and its sequelae are one of the most prevalent complications of MRM, occurring in 15%-60% of patients [3]. Seroma is an exudative fluid collection that develops postoperatively after MRM in the dead space beneath the mastectomy flaps and the axilla. Most seromas are asymptomatic and resolve in a month, but 15% become symptomatic, increasing postoperative morbidity, infection risk, flap complications, delayed healing, patient discomfort, prolonged hospital stays, and delayed adjuvant treatment [3,4]. Even though seroma is the most frequent side effect of mastectomy with axillary dissection, little is known about its etiopathogenesis, which is thought to be multifactorial [5].

Managing seromas remains challenging, with no universally effective protocol despite years of clinical experience. A decrease in postoperative seroma depends on the obliteration of the dead space by external compression, a closed suction drain obliteration of space, fibrin sealants, or suture flap fixation to the chest wall [4,5]. Flap fixation, also known as quilting or tissue anchoring, has emerged as an effective surgical technique to minimize seroma formation in various clinical trials and studies [6,7]. Axillary exclusion is a technique aimed at obliterating dead space after axillary clearance. By reducing the potential space for fluid collection underneath flaps and axillae, flap fixation with axillary exclusion promotes better adhesion between the layers and improves postoperative outcomes [8].

This study evaluated the efficacy of flap fixation with axillary exclusion by interrupted fine sutures, which would effectively reduce drain output and seroma formation while minimizing cosmesis.

## Materials and methods

Aims and objectives

The study aims to evaluate the efficacy of flap fixation with axillary exclusion by interrupted fine sutures, which would effectively reduce drain output and seroma formation.

The primary objective of this study is to evaluate the effect of flap fixation with the axillary exclusion in reducing seroma formation compared to conventional closure in MRM patients. The secondary objective is to compare postoperative morbidity in flap fixation with the axillary exclusion group compared to the conventional closure group regarding pain, surgical site infection (SSI), and flap necrosis.

Study design and setting 

After ethical clearance, a prospective comparative study was conducted in the General Surgery Department in a tertiary care teaching hospital from April 1, 2019, to March 31, 2020 (IHEC/LOP/2019/MD0054).

Inclusion criteria

We included adult female patients with a diagnosis of breast carcinoma of more than 18 years of age who were advised of MRM as surgical treatment.

Exclusion criteria

We excluded patients who needed immediate breast reconstruction or conservative breast surgery and those with a history of previous axillary surgery.

Sample-size estimation

The sample size was 72 patients (36 in each group), calculated with a margin of error of 5% and a confidence interval of 95% with 80% power and calculated using Gpower statistical software 3 1.9.7 version.

Interventions

Patients were allocated sequentially and alternately into two groups Study group (flap fixation with the axillary exclusion) and Control group (conventional closure), with 36 assigned to each group per the inclusion and exclusion criteria.

*Study Group* *(n = 36)*

**Figure 1:**
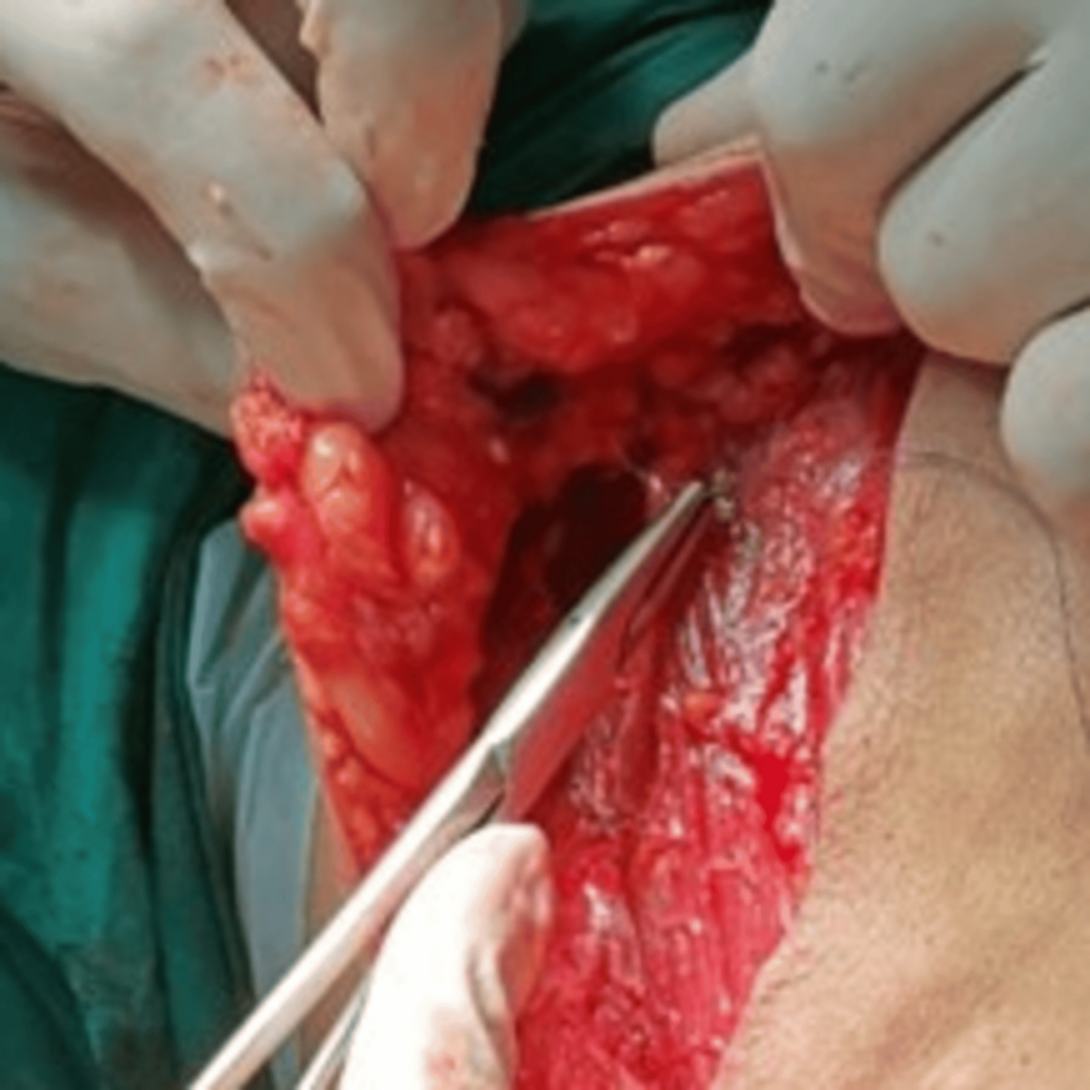
Inferior flap fixation in MRM (over chest wall using absorbable suture) MRM: Modified radical mastectomy

**Figure 2:**
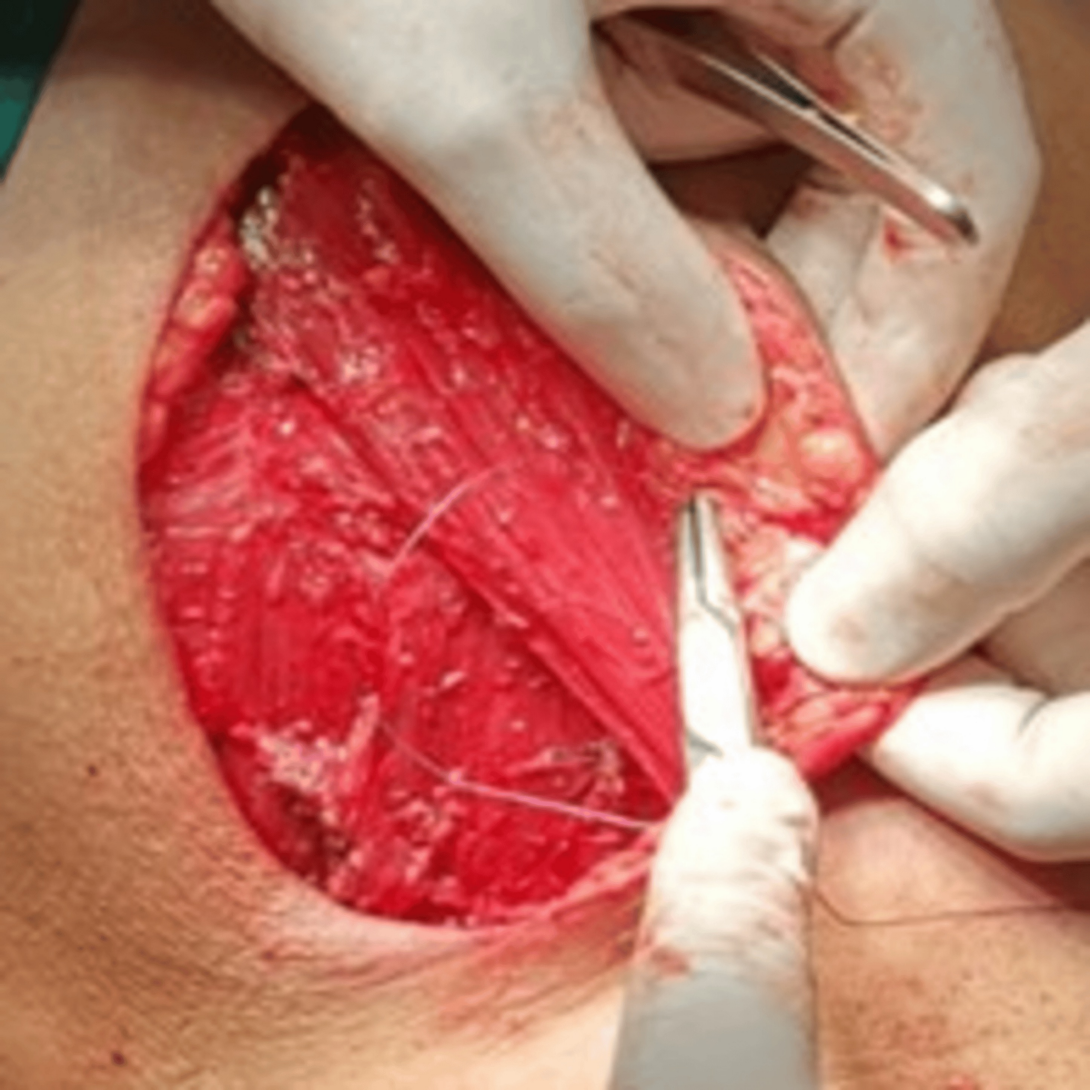
Superior flap fixation in MRM (over pectoralis major using absorbable suture) MRM: Modified radical mastectomy

In the study group, MRM was performed as per standard protocol by using monopolar and bipolar cautery as needed. During the closure of mastectomy flaps, the subcutaneous tissues of the superior and inferior skin flaps were fixed to the underlying pectoralis major muscles by four to five interrupted sutures with 3-0 polyglactin absorbable sutures 3-4 cm apart in each flap (Figures 1, 2). The axillary exclusion was done by approximating the pectoralis major with the pectoralis minor muscle and the lateral wall of the axilla to the fascia of the serratus anterior muscle by interrupted absorbable 3-0 polyglactin sutures. Care was taken to prevent dimpling of the skin. A single negative-pressure closed-suction drainage system with two draining catheters (14 F) was placed, with one catheter tip in the axillary area and the other catheter tip underneath the superior flap. Both draining catheters were brought out and fixed to the skin of the inferior flap, 2-3 cm below in the anterior axillary line, and connected to the suction chamber of the drainage system. The skin edges were sutured in a single layer using 3-0 poliglecaprone absorbable sutures.

Control Group (n = 36)

In the control group, MRM was performed as per standard protocol by using monopolar and bipolar cautery as needed. Negative pressure closed suction drains were placed underneath the mastectomy flaps and axilla, as mentioned in the study group. The mastectomy flaps were approximated in two layers. The subcutaneous layers were sutured with 3-0 polyglactin absorbable interrupted sutures, and skin edges were sutured in a single layer using 3-0 poliglecaprone absorbable sutures.

Outcome measures

Primary Outcomes

Postoperatively, the total drain output, the day of drain removal, and the incidence of seroma were noted and recorded in the case record form.

The operative definition of seroma is abnormal collection of fluid beneath the mastectomy flaps that were symptomatic and needed treatment with aspiration or or surgical intervention (Grade 2 and 3 CTAE classification). As per the Criteria for Adverse Events Classification (CTCAE) version 4.0, seroma is categorized into three grades. Grade 1 seroma is asymptomatic and does not require intervention, Grade 2 seroma is symptomatic and can be treated with aspiration, and Grade 3 seroma is symptomatic and requires surgical and radiological intervention [6].

Secondary Outcomes

Postoperative complications like pain (measured by visual analog scale (VAS)), SSIs, and flap necrosis were also noted and recorded in the case record form.

Data collection

The tumor characteristics and operation-related factors were recorded in the study and control group patients. Operative time and any intraoperative complications were recorded. The amount and color of drained fluid were recorded daily. The drains were removed when the amount became less than 30 cc. Patients were evaluated postoperatively regarding daily drain volume, total drain volume, postoperative day of drain removal, seroma, and wound complications.

Local chest wall ultrasound over the flaps and axilla was done two weeks after drain removal to document or exclude the presence of any collections. Incidence of seroma formation, total drain output, and the day the drain was removed were recorded for all patients and compared to the control group of patients undergoing conventional MRM in the tertiary-care teaching hospital.

**Figure 3:**
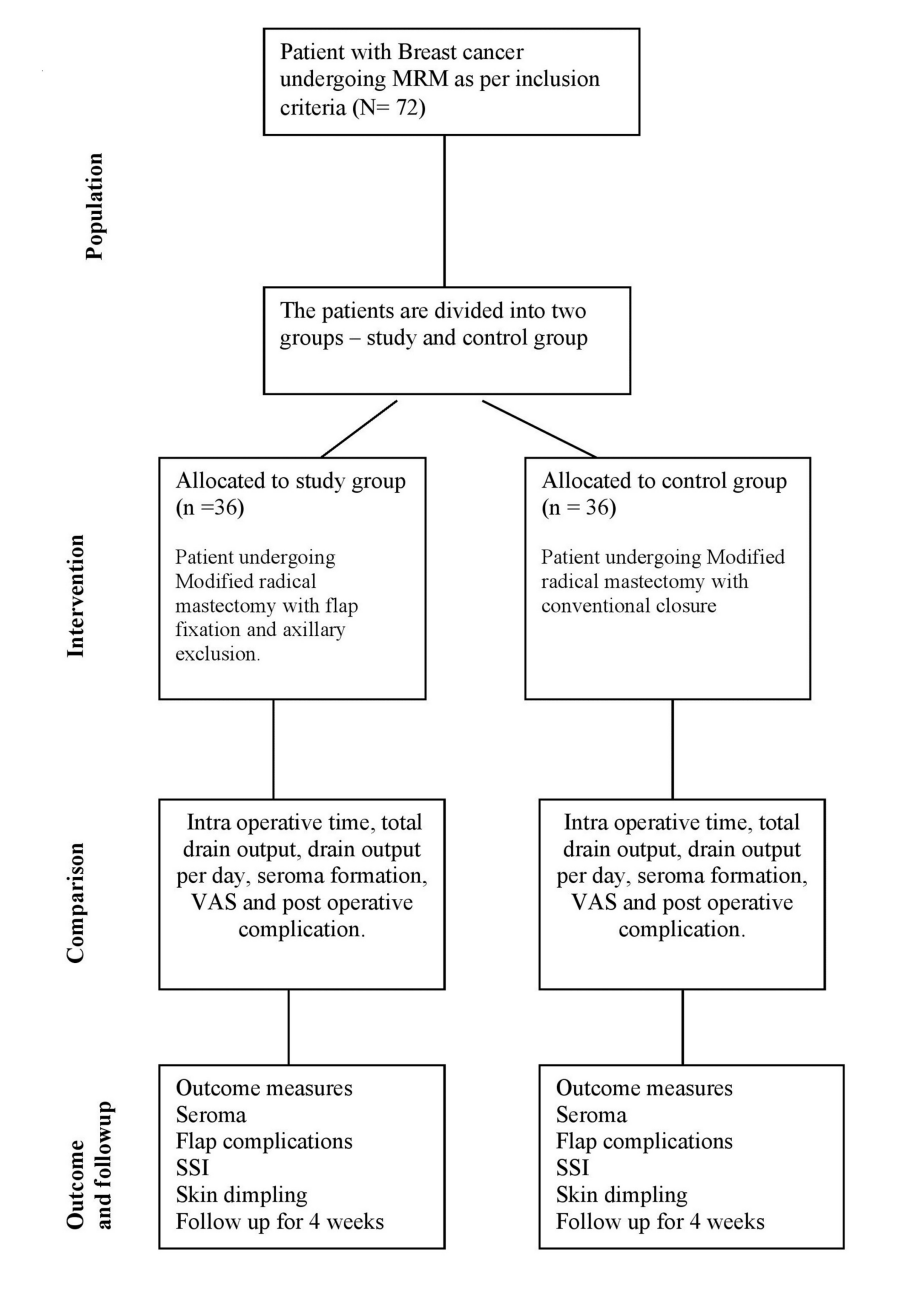
Consort diagram of the study

Statistical analysis

Data were analyzed using SPSS software. Continuous variables were compared using the t-test, and categorical variables were analyzed with the chi-square test. Data were represented in tables and graphs; continuous quantitative variables, e.g., age, were expressed as the mean ± SD and median (range); and categorical qualitative variables were expressed as absolute frequencies (number) and relative frequencies (percentage). Suitable statistical tests of significance were used after checking for normality. The results were considered statistically significant when the significant probability was less than 0.05 (P < 0.05), and a P-value ≥ 0.05 was considered statistically insignificant (NS).

## Results

All the patients included in this study were women. The mean age of patients in the study and control groups was 48.78 years and 49.11 years, respectively, with no significant difference observed. The prevalence of comorbidities like diabetes was comparable between the two groups. Additionally, the average BMI and comorbidities of the study and control groups showed no significant variation. The distribution of patients as per the clinical staging was mainly in stages 2 and 3 and was broadly comparable in both groups. The number of lymph nodes retrieved and level of axillary lymph node dissection and lymphovascular invasion on histopathological evaluation were not statistically significant between the study and control groups, as shown in Table 1

The outcome measures, such as operative time, drain volume (total and per day), and drain removal day, were recorded in Table 1. There was no statistically significant difference in operating time between the flap fixation group (115.83 minutes) and the conventional closure group (112.92 minutes), as indicated by a P-value of 0.509. This suggests that flap fixation with axillary exclusion does not substantially increase the duration of surgery. The flap fixation group had a significantly lower total drain volume (306.67 mL) compared to the conventional closure group (531.11 mL), with a P-value < 0.01. This demonstrates that flap fixation with axillary exclusion effectively reduces the accumulation of postoperative fluid, a critical factor in preventing seroma formation. The average daily drain volume was significantly lower in the flap fixation group (73.12 mL/day) than in the control group (83.17 mL/day), study group. This reinforces the benefit of flap fixation with axillary exclusion in minimizing fluid output per day, facilitating faster recovery. Patients in the flap fixation group had their drains removed earlier (average of 4.00 days) compared to the control group (6.25 days), with a significant P-value of <0.001. Early drain removal in flap fixation with axillary exclusion group reduces the hospital stay and improves patient comfort.

**Table 1:** Comparision of operative time, drain volume, drain volume per day and drain removal day

Characteristics	Study group (Mean)	Control group (Mean)	t-value	P-value (0.005)
Operating time	115.83	112.92	0.67	0.509
Drain volume (total)	306.67	531.11	4.23	<0.01
Drain volume (per day)	73.12	83.17	2.01	0.044
Drain removal day	4.00	6.25	5.12	<0.001

Seroma incidence was recorded in Table 2.

The total seroma incidence (clinical and radiological) is significantly lower (P < 0.001) in the flap fixation and axillary exclusion group (study group) (n = 4, 11.1%) compared to the control group (n = 15, 41.7%). Grade 1 seroma (n = 3, 8.3% versus n = 8, 22.2%) and Grade 2 seroma (n = 1, 2.8% versus n = 6, 16.7%) were significantly lower (P < 0.001) in the study group, highlighting that flap fixation with axillary exclusion effectively reduces seroma formation.

**Table 2:** Seroma incidence in study and control group

Characteristics	Study group n (%)	Control group n (%)	Chi-square value	P-value (0.05)
Total seroma (clinical and radiological)	4 (11.1%)	15 (41.7)	15.02	<0.001
Grade 1	3 (8.3%)	8 (22.2)	10.27	<0.001
Grade 2	1 (2.8%)	6 (16.7)	7.42	<0.001
Grade 3	0	1 (2.8%)	1.25	0.264

Postoperative complications, such as SSI, flap necrosis, and skin dimpling, are recorded in Table 3. SSI occurred in three patients (8.33%) in the flap fixation group and four patients (11.11%) in the conventional closure group (P = 0.72), showing no significant difference, which indicates that SSI is not increased by flap fixation with axillary exclusion. Flap necrosis was found in two cases (5.5%) in both groups (p -1.0), with no significant difference, thus indicating that flap fixation with axillary exclusion does not increase flap necrosis. Although skin dimpling was lower in the flap fixation group (n = 4, 11.11%) compared to the control group (n = 8, 22.22%), it was not statistically significant.

**Table 3:** Comparison of postoperative complications: SSI, flap necrosis and skin dimpling SSI: Surgical site infection

Characteristics	Study group n (%)	Control group n (%)	Chi-square value	P-value (0.05)
SSI	3 (8.33)	4 (11.11)	0.13	0.72
Flap necrosis	2 (5.5)	2 (5.5)	0.00	1
Skin dimpling	4 (11.11)	8 (22.22)	1.51	0.217

The mean VAS score (2.78) for pain was the same in both groups, with a non-significant P-value (0.916), indicating that there was no associated additional pain with the flap fixation and axillary exclusion group compared to the conventional closure group. Hence, the flap fixation technique does not compromise the patient's comfort.

## Discussion

Seroma formation after MRM cannot be avoided because of dead space and axillary lymph node dissection. Several studies and clinical trials have shown suturing mastectomy flaps to the pectoralis major and lateral chest wall with interrupted or continuous absorbable sutures as an effective method for minimizing dead space. Chilson et al. presented the concept of flap fixation for reducing seroma formation in 1992 [9]. The first multi-center, double-blind, randomized controlled trial (Seroma Reduction After Mastectomy (SAM) trial) recommended suture flap fixation in mastectomy for the reduction of seroma [10]. Foulan et al. found a reduction in seroma of about 21.7%; similarly, Bhagchandani et al. and Garzali et al. reported a reduction of approximately 34.3% and 30% using the flap fixation method [2,11,12].

Most studies show statistically significant reductions in drain output and seroma formation in patients undergoing MRM with flap fixation. However, the reduction is much more pronounced in the flap fixation when combined with the axillary exclusion group, indicating that isolating the axilla is key to minimizing postoperative fluid accumulation. The axillary exclusion is achieved by securing the pectoralis major and minor muscles to reduce axillary dead space and separate it from the mastectomy cavity [13,14]. van Bemmel et al. also recommended that closing the axillary dead space along with flap fixation had a cumulative effect in reducing the seroma formation [15]. Chand et al. and Khater et al. found a reduction in seroma of approximately 48% and 58%, respectively [14,16]. Similarly, Mohamed et al. and Jain et al. reported a reduction of 18.2% and 26.5%, respectively, using flap fixation with axillary exclusion [17,18]. In our study, flap fixation with axillary exclusion effectively reduces clinically significant seroma (Grades 2, 3) from 19.4% in the conventional flap closure group to 2.8%, along with reduced dimpling, similar to Myint et al. [19].

In our study, flap fixation with axillary exclusion in MRM significantly reduced the average daily and total drain volumes by 45.2% compared to conventional closure, allowing for earlier drain removal (4 versus 6.25 days, P < 0.001). As a result, patients experienced shorter hospital stays, lower rates of seroma formation, and fewer aspirations, leading to faster recovery. Similar results were seen by Irfan et al.,with a reduction of drain output of 44.5% and 49.1% with a statistically significant P-value [8].

Velotti et al.'s meta-analysis found a cumulative SSI rate of 9.7% in the conventional closure group and 8.6% in the flap fixation group, with no statistically significant difference [20]. A similar result was observed in our study, with a decrease in SSI from 11.11% (conventional closure group) to 8.33% (flap fixation group) with no significant difference (P = 0.72), indicating that flap fixation with axillary exclusion does not increase the risk of SSI and flap necrosis.

Moreover, flap fixation with continuous suturing causes skin dimpling and puckering, which can be reduced using fine and interrupted anchoring sutures, as seen by Myint et al. and in our study [19]. Although the cosmetic appearance after fixation was reported to be satisfactory, a drawback of this technique is the additional operating time required. Reports indicate that the procedure can extend the surgery by up to 20 minutes [14]. In our study, no significant difference was noted regarding operative time. Future research should focus on the best ways of reducing seroma by combining proven methods.

Limitations of the study

The study included 72 patients, with 36 participants each in study and control group, which limits the extent to which the findings can be generalized to a wider population. To identify the smaller differences for reaching more statistically significant conclusions, a larger sample size is needed. Another limitation of the study is the study design, which is a prospective and comparative selection bias. Randomization will minimize the confounding variables with better level of evidence.

## Conclusions

Flap fixation with axillary exclusion is a valuable technique in minimizing seroma formation and eliminating mechanical dead space after MRM. This study supports its routine adoption in surgical practice, as it enhances postoperative recovery and reduces patient morbidity. While drains remain necessary post MRM, flap fixation with axillary exclusion offers an effective method to mitigate seroma-related complications and improve patient outcomes. Given that flap fixation with axillary exclusion after mastectomy significantly reduces drainage duration, seroma formation, and the need for aspiration, their integration into standard mastectomy procedures is highly recommended.
